# Severe ARDS Complicated by Active Pulmonary Tuberculosis and Recurrent Nosocomial Infections: Therapeutic Challenges and Clinical Outcomes

**DOI:** 10.3390/life15071068

**Published:** 2025-07-04

**Authors:** Wei-Hung Chang, Yi-Ting Wang, Ting-Yu Hu, Li-Kuo Kuo

**Affiliations:** 1 Department of Critical Care Medicine, MacKay Memorial Hospital, Taipei 104, Taiwan; peacejaycool@gmail.com (W.-H.C.); cherry.wang822@gmail.com (Y.-T.W.);; 2 Department of Medicine, Mackay Medical College, New Taipei City 252, Taiwan

**Keywords:** ARDS, tuberculosis, ventilator-associated pneumonia, nosocomial infection, gastrointestinal bleeding, polio, bronchoscopy, critical care, case report

## Abstract

**Background:** Acute respiratory distress syndrome (ARDS) secondary to tuberculosis (TB) is rare and associated with high mortality. Management is further complicated by comorbidities and ICU-related complications. **Methods:** We report a 43-year-old woman with post-polio sequelae and uncontrolled diabetes who developed ARDS due to pulmonary TB, complicated by recurrent nosocomial infections and gastrointestinal bleeding. Early bronchoscopy and GeneXpert MTB/RIF PCR were performed on ICU Day 2, enabling anti-TB therapy initiation by ICU Day 3. The patient received lung-protective ventilation, prone positioning, tailored antibiotics, and multidisciplinary care. **Results:** The patient’s clinical course was complicated by two episodes of ventilator-associated pneumonia and gastrointestinal bleeding, but with individualized management, she achieved ventilator weaning and functional recovery. **Conclusions:** Early TB recognition in ARDS is crucial. Multidisciplinary ICU management, including prudent steroid use, improves outcomes.

## 1. Introduction

Acute respiratory distress syndrome (ARDS) is a severe, life-threatening condition marked by acute onset, profound hypoxemia, and diffuse pulmonary infiltrates not fully explained by cardiac failure or volume overload [1]. Globally, ARDS remains a major cause of morbidity and mortality among critically ill patients, accounting for approximately 10% of all ICU admissions [2,3]. Despite advances in supportive care, mortality rates remain high, particularly for patients with severe ARDS, defined as a PaO_2_/FiO_2_ ratio less than 100 mmHg under positive pressure ventilation according to the Berlin definition [3].

Recent international cohort studies, including the LUNG SAFE study, have highlighted persistent heterogeneity in ARDS management and outcomes across regions, emphasizing the need for context-specific diagnostic and therapeutic strategies [4,5]. In East Asia, including Taiwan, population-based registries indicate that infectious etiologies—especially bacterial pneumonia and tuberculosis—remain prominent causes of ARDS, but the clinical recognition of TB-ARDS is often delayed [6,7].

Tuberculosis (TB) is a rarely reported precipitant, accounting for 1–3% of ARDS ICU admissions, yet associated with exceedingly high mortality [8,9]. TB-ARDS patients may present without typical respiratory or constitutional symptoms, and up to 40% of cases manifest as so-called “atypical ARDS,” further complicating prompt diagnosis and management [10].

The pathophysiology of TB-ARDS involves diffuse alveolar damage, intense cytokine-mediated inflammation, and sometimes a hematogenous spread of Mycobacterium tuberculosis (miliary TB) [11]. Recent advances in molecular diagnostics, especially GeneXpert MTB/RIF PCR (reported sensitivity of ~89% and specificity of ~99% for pulmonary TB [12]) have reduced the median diagnostic delay from weeks to days, which is crucial given that early anti-TB therapy initiation (within 72 h) significantly improves survival [12,13]. Nevertheless, real-world evidence for optimal management, especially in multi-comorbid or immunocompromised patients, is limited [14].

Management is further complicated by ventilator-associated pneumonia (VAP), gastrointestinal bleeding, prolonged ventilatory support, and the need for immunomodulation. The overlap of post-polio sequelae and poorly controlled diabetes adds additional barriers to successful ICU management and rehabilitation [15,16].

Case reports and small case series suggest that a multidisciplinary approach—including respiratory therapy, infectious disease consultation, early rehabilitation, and intensive glycemic management—may be critical for optimizing outcomes in TB-ARDS [17,18]. This report details a severe TB-ARDS case with multiple complications, illustrating both the diagnostic value of early bronchoscopy and PCR-based testing, and the therapeutic importance of individualized, protocolized ICU management.

## 2. Materials and Methods (Case Description)

### 2.1. Patient History and Initial Presentation

A 43-year-old Taiwanese woman, with a known history of childhood poliomyelitis resulting in right lower limb weakness, presented to the emergency department with three days of malaise, worsening dyspnea, productive cough, and confusion. She denied previous TB diagnosis, recent travel, or known TB contacts. Her medical history was also significant for poorly controlled type 2 diabetes mellitus (most recent HbA1c, 10.4%), hypertension, and obesity (BMI, 28 kg/m^2^).

On arrival, the patient was febrile (37.2 °C), tachycardic (HR, 103), tachypneic (RR, 28), hypotensive (BP, 94/61 mmHg), and hypoxic (SpO_2_, 88% on room air). The Glasgow Coma Scale was E2V1M5. Arterial blood gas analysis revealed a pH of 6.81, PaCO_2_ of 28 mmHg, HCO_3_ of 5 mmol/L, lactate of 6.8 mmol/L, glucose of 538 mg/dL, and serum ketones of 4.3 mmol/L, consistent with severe diabetic ketoacidosis (DKA) and lactic acidosis. Other initial labs demonstrated leukocytosis (WBC, 21,000/μL), a CRP of 24 mg/dL, and acute kidney injury. Shortly after arrival, the patient developed cardiac arrest with an initial rhythm of asystole. She underwent 12 min of advanced cardiac life support (ACLS), during which two cycles of CPR and epinephrine were performed. A brief episode of pulseless ventricular tachycardia occurred, prompting the administration of amiodarone (300 mg IV). A return of spontaneous circulation (ROSC) was achieved, and she was intubated, stabilized with vasopressors, and transferred to the intensive care unit (ICU). Post resuscitation, targeted temperature management was not performed, due to rapid neurological recovery and ongoing hemodynamic instability. Standard temperature and neurologic monitoring were continued.

### 2.2. ICU Course and Interventions

**Figure 1:**
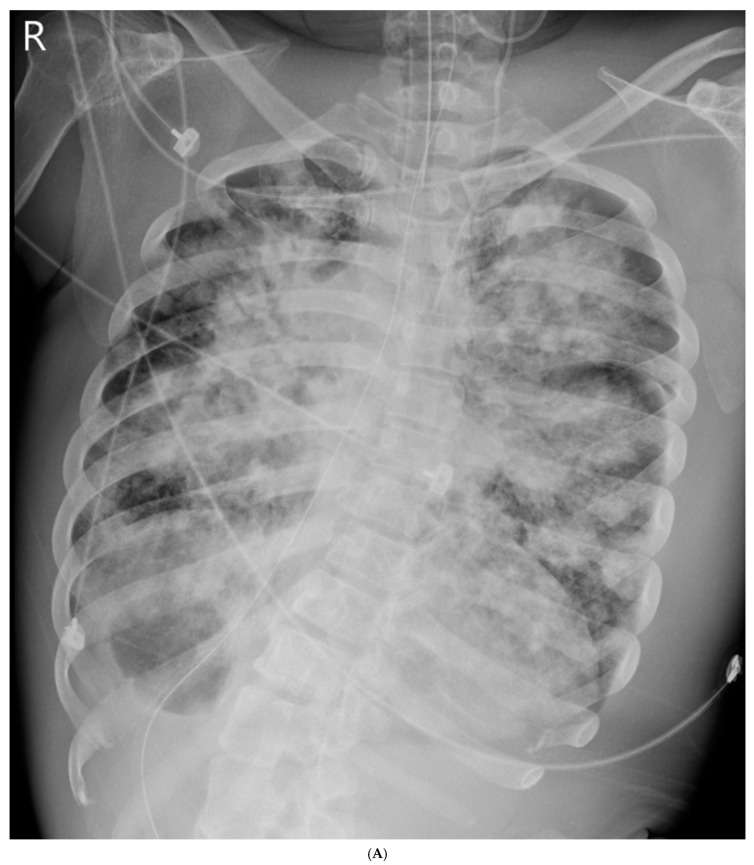

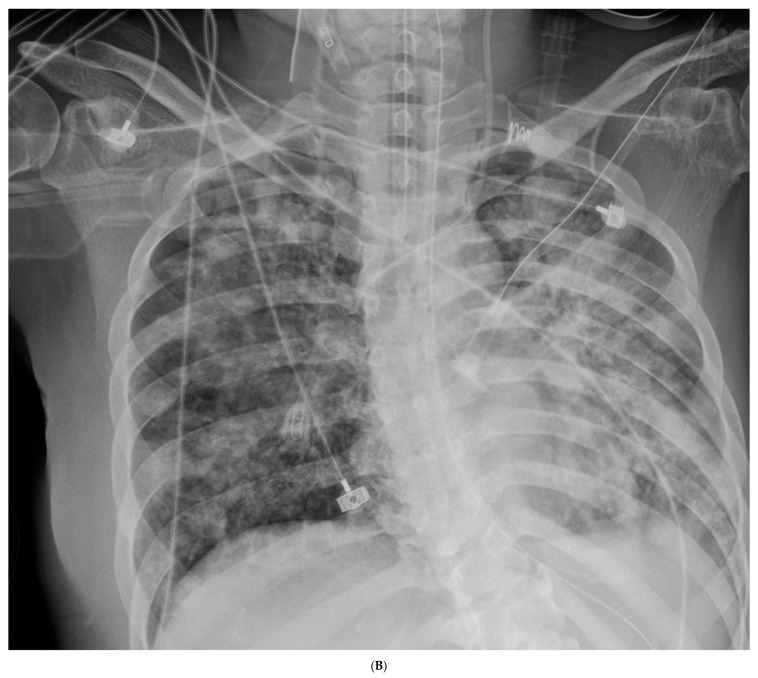

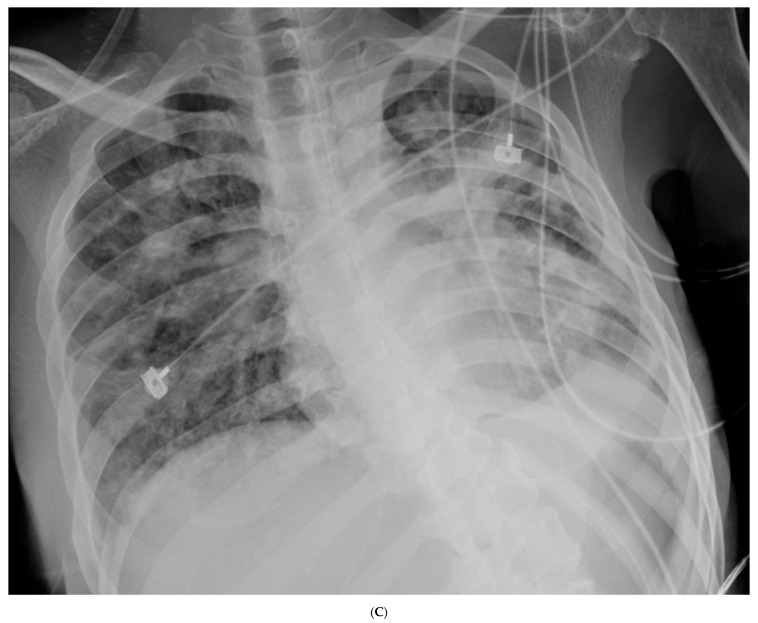
Chest radiograph on ICU Day 1 showing bilateral diffuse infiltrates. (**A**) On ICU Day 0, at admission, showing diffuse bilateral alveolar infiltrates consistent with severe ARDS. (**B**) On ICU Day 10, after prone positioning and anti-TB therapy initiation, showing persistent infiltrates but some improvement in aeration. (**C**) On ICU Day 50, prior to ICU discharge, showing substantial resolution of infiltrates.

Upon ICU admission (Day 0), chest radiography revealed diffuse bilateral alveolar infiltrates with no evidence of cavitation (Figure 1). Mechanical ventilation was initiated in volume-controlled mode: tidal volume, 6 mL/kg predicted body weight; PEEP, 12–14 cmH_2_O; and FiO_2_, 1.0. Sedation was maintained with propofol and fentanyl. Vasopressor support (norepinephrine and vasopressin) was used to maintain mean arterial pressure. Acute kidney injury was managed with intravenous fluids and vasopressor support. Renal replacement therapy was not required, and renal function returned to baseline during recovery. Empirical antibiotics (ceftriaxone and azithromycin) were started for severe community-acquired pneumonia. Early enteral nutrition was provided within 24 h of ICU admission. Laboratory and radiographic findings during the initial ICU course are shown in Table 1.

Tuberculosis is a contagious airborne disease. During the patient’s management in the emergency department—including advanced cardiac life support (ACLS) and intubation—airborne precautions were strictly implemented in accordance with CDC and hospital infection control guidelines. All staff involved in resuscitation and airway management wore N95 respirators, eye protection, gowns, and gloves. After ICU admission, the patient was managed in a negative-pressure isolation room. All aerosol-generating procedures were performed under full airborne precautions. Importantly, there were no cases of occupational TB transmission among the medical staff, as confirmed by routine post-exposure surveillance.

On ICU Day 2, flexible bronchoscopy was performed, revealing copious yellowish purulent secretions in both lower lobes. Bronchoalveolar lavage (BAL) samples were sent for Gram staining, bacterial culture, fungal culture, acid-fast bacilli (AFB) smear, GeneXpert MTB/RIF PCR, and viral PCR panels including SARS-CoV-2, all of which were negative. The initial results for bacterial and fungal cultures were negative. On ICU Day 3, GeneXpert PCR was positive for Mycobacterium tuberculosis (rifampin-sensitive), and the AFB smear was positive as well. First-line anti-TB therapy was initiated: isoniazid (300 mg), rifampin (600 mg), ethambutol (1200 mg), and pyrazinamide (1500 mg). Steroids were withheld initially to avoid immunosuppression during the early phase of TB treatment; however, methylprednisolone (40 mg/day) was initiated on ICU Day 6 due to persistent severe hypoxemia (PaO_2_/FiO_2_ < 100 mmHg) after anti-TB therapy, in line with international ARDS guidelines [19].

Despite lung-protective ventilation, the patient’s PaO_2_/FiO_2_ ratio remained below 70 mmHg. Prone positioning was implemented from ICU Day 5 to Day 8 (16 h per session) in conjunction with cisatracurium for neuromuscular blockade and deep sedation. Following three days of prone positioning, oxygenation improved, with PaO_2_/FiO_2_ rising to 150 mmHg by ICU Day 10. Passive and active-assisted range of motion, as well as early mobilization (including sitting at the edge of the bed), were started as soon as hemodynamics permitted, in collaboration with rehabilitation specialists and nursing staff. Glycemic control was maintained with continuous insulin infusion protocols.

On ICU Day 10, the patient developed fever, increased sputum production, and a new right lower lobe infiltrate on chest X-ray. BAL culture identified Sphingomonas paucimobilis, leading to a change in antibiotics to piperacillin-tazobactam with adjunctive inhaled amikacin. On ICU Day 14, the patient developed gastrointestinal bleeding, with coffee-ground material in the nasogastric tube and a decrease in hemoglobin from 8.1 to 7.0 g/dL. Upper gastrointestinal endoscopy revealed multiple gastric erosions but no active bleeding. The patient was on intravenous pantoprazole for stress ulcer prophylaxis prior to GI bleeding; following the event, therapy was escalated and endoscopic evaluation was performed.

An attempted spontaneous breathing trial (SBT) on ICU Day 15 resulted in extubation failure due to poor airway protection, necessitating reintubation. Early tracheostomy was performed on ICU Day 21 to facilitate airway clearance, reduce ventilator-associated complications, and allow for further weaning. On ICU Day 28, a second episode of ventilator-associated pneumonia occurred, with a BAL culture isolating Acinetobacter baumannii susceptible to colistin and tigecycline; colistin (loading dose 9 MIU, then 4.5 MIU every 12 h) and tigecycline (100 mg loading, then 50 mg every 12 h) were administered according to MIC results and an institutional antibiogram.

With ongoing anti-TB therapy, optimal ventilator management, and multidisciplinary ICU support, the patient’s oxygenation and infection parameters steadily improved. By ICU Day 45, FiO_2_ had been reduced to 0.35 and PEEP to 6 cmH_2_O. The patient was fully liberated from the ventilator, and tracheostomy decannulation was achieved by ICU Day 50. Serial sputum AFB smears and cultures were negative by ICU Day 48. She was transferred to the respiratory ward on ICU Day 54 for continued anti-TB therapy and rehabilitation.

Multiple sets of blood cultures were obtained during the ICU stay, all of which were negative for bacterial growth. Transthoracic echocardiography performed after ROSC revealed preserved left ventricular systolic function with no evidence of significant structural heart disease. Key laboratory trends and detailed medication regimens (including steroid doses, anti-TB, and antibiotics), as well as clinical interventions, are summarized in Table 1. No renal replacement therapy was required throughout the ICU course. The overall clinical course and key interventions are visually summarized in Table 2.

## 3. Results

The patient survived a 54-day ICU course complicated by severe ARDS, two episodes of VAP, gastrointestinal bleeding, and multi-organ dysfunction. Throughout her stay, a high index of suspicion for TB, the use of early molecular diagnostics, and coordinated ICU care were critical to her recovery. Sputum AFB smears and cultures converted to negative within seven weeks of anti-TB therapy. After successful ventilator weaning and tracheostomy decannulation, the patient was able to participate in progressive rehabilitation and regained her pre-hospital functional status at the three-month follow-up. No recurrence of infection or bleeding was observed during a three-month follow-up after ICU discharge. Her glycemic control and renal function returned to baseline.

## 4. Discussion

ARDS resulting from pulmonary TB remains a rare but highly lethal clinical entity. Delays in diagnosis are common due to the overlapping features with other causes of ARDS and the atypical presentation of TB in critically ill patients [20]. This case illustrates how the early use of bronchoscopy and rapid molecular testing with GeneXpert MTB/RIF PCR facilitated prompt diagnosis and targeted therapy, which has been shown to improve survival if started within 72 h of ARDS onset [21].

Recent meta-analyses confirm that early anti-TB therapy is the most important modifiable predictor of mortality in TB-ARDS, with delays over 72 h resulting in a doubling of the mortality risk [22,23]. Importantly, studies from high-TB-burden countries emphasize the value of early empirical anti-TB therapy in cases of rapidly progressive ARDS of unclear etiology [24]. In Taiwan and other Asia-Pacific settings, the integration of molecular diagnostics into ICU protocols has been linked to increased survival and a shorter hospital length of stay [25].

Lung-protective ventilation strategies and protocolized prone positioning were key interventions in managing refractory hypoxemia, consistent with international ARDS guidelines [19,26]. Adjunctive therapies, such as neuromuscular blockade and higher PEEP, remain debated in TB-ARDS but may be reasonable in severe hypoxemia or when complicated by neuromuscular weakness [27]. The timing and dosing of corticosteroids in TB-ARDS remain controversial, with differing practices in non-HIV TB, miliary TB, and severe ARDS. Evidence remains limited, and expert consensus varies [28,29].

The management of VAP and other nosocomial infections is especially complex in the context of TB-ARDS, given the need for prolonged mechanical ventilation and the increased risk for multidrug-resistant organisms [30]. Multidisciplinary input from pharmacists, infection control, and respiratory therapy is critical in guiding antimicrobial stewardship and optimizing patient outcomes [31]. The early initiation of enteral nutrition, strict glycemic control, and aggressive physical therapy further contribute to successful weaning and functional recovery [32,33].

Gastrointestinal bleeding, while relatively underreported in the ARDS literature, is a major cause of morbidity in prolonged ICU courses, especially when compounded by coagulopathy, vasopressors, and stress-related mucosal injury [34]. Acid suppression, early endoscopic evaluation, and prompt transfusion, as performed in this case, are consistent with best practices.

Unique to our case is the presence of post-polio sequelae, which is associated with a reduced ventilatory reserve and greater risk for ICU-acquired weakness and weaning failure [35]. There is growing evidence supporting early and intensive physical medicine and rehabilitation in critically ill neuromuscular patients, with improved outcomes in both ICU and long-term follow-up [36,37].

This case adds to the growing body of literature emphasizing the need for high clinical suspicion for TB in ARDS of unexplained etiology, particularly in TB-endemic regions or in immunocompromised hosts [38,39]. The implementation of standardized ARDS management bundles that include molecular TB testing and early rehab should be strongly considered in future multicenter studies. Further research into individualized immunomodulation and rehabilitation protocols tailored to patients with neuromuscular comorbidities is warranted.

The diagnosis of TB-ARDS in this case was complicated by several atypical features and overlapping potential etiologies. Although active Mycobacterium tuberculosis infection was confirmed early, and the clinical improvement correlated with anti-TB therapy, the radiographic evolution did not show classic findings such as cavitation, granuloma formation, or fibrotic changes either during the acute phase or on follow-up imaging. This raises the question of whether ARDS was directly attributable to TB, or if other factors such as severe diabetic ketoacidosis, post-cardiac arrest circulatory failure, or early ventilator-associated pneumonia (notably Sphingomonas paucimobilis) played contributory roles. However, the patient’s bilateral diffuse infiltrates were already present at admission, before VAP could have developed. There was no echocardiographic evidence of pre-existing heart failure. Severe metabolic derangements and circulatory shock are known risk factors for ARDS, especially in immunocompromised patients. Therefore, it is likely that ARDS in this patient was multifactorial, with TB infection acting as a key trigger in a susceptible host with poorly controlled diabetes and acute metabolic decompensation. Such diagnostic complexity highlights the need for broad differential consideration and comprehensive assessment in ARDS of unclear etiology. Although Sphingomonas paucimobilis was isolated in the BAL fluid on ICU Day 2, this finding likely represented a secondary infection rather than the primary cause of ARDS. The patient presented with diffuse bilateral pulmonary infiltrates and severe hypoxemia on admission, prior to the identification of Sphingomonas. Moreover, diabetes management was promptly initiated upon admission. Therefore, the timing and clinical course support that ARDS was primarily precipitated by active tuberculosis and metabolic decompensation, while Sphingomonas represented a subsequent ventilator-associated pneumonia.

## 5. Conclusions

TB-associated ARDS presents complex diagnostic and management challenges, particularly in patients with multiple comorbidities and ICU complications. Early recognition, rapid diagnostic confirmation with molecular methods, prompt anti-TB therapy, and vigilant monitoring for nosocomial infections and gastrointestinal bleeding are essential for optimizing outcomes. A coordinated multidisciplinary approach, including tailored ventilatory strategies and early rehabilitation, can lead to survival and functional recovery even in high-risk TB-ARDS patients.

## Figures and Tables

**Table 1:** Integrated timeline of key clinical events, laboratory trends, medication dosages, and therapeutic interventions during the ICU course. MPD: methylprednisolone; HREZ: isoniazid, rifampin, ethambutol, pyrazinamide; RRT: renal replacement therapy; VAP: ventilator-associated pneumonia; GI: gastrointestinal; TGC: tigecycline.

ICU Day	Key Clinical Events	pH	PaO_2_/FiO_2_ (mmHg)	CRP (mg/dL)	Hb (g/dL)	Steroid (mg/d)	Anti-TB	Antibiotics	RRT
0	Intubation, prone, septic shock	6.81	60 (FiO_2_ 1.0)	15.5	8.1	None	None	CTX/AZI	No
1		7.30	80	14.1	7.1	None	None	CTX/AZI	No
3	TB confirmed via PCR, HREZ start	7.35	150	10.3	10.7	None	HREZ	CTX/AZI	No
6	MPD 40 mg/d start (steroid)	7.30	70	13.1	10.3	MPD 40	HREZ	CTX/AZI	No
7	Improved O_2_, start PT/rehab	7.45	200	2.6	9.4	MPD 40	HREZ	CTX/AZI	No
10	VAP #1, change to TZP+Amikacin	7.41	150	2.2	8.1	MPD 40	HREZ	TZP+Amikacin (inh)	No
14	GI bleeding, EGD, transfusion	7.45	250	1.2	7.7	MPD 40	HREZ	TZP+Amikacin (inh)	No
15	Extubation fail, re-intubation	7.10	120	1.5	10.7	MPD 32	HREZ	TZP	No
21	Tracheostomy	7.38	300	0.8	8.5	MPD 16	HREZ	TZP	No
28	VAP #2 (AB), change to Colistin/TGC	7.44	320	0.6	8.0	MPD 8	HREZ	Colistin/TGC	No
45	Weaning, off steroid/abx	7.47	350	0.5	9.3	Off	HREZ	Off	No
54	Transfer to ward, rehab	7.45	400 (RA)	0.5	11.0	Off	HREZ	Off	No

**Table 2:** Simplified clinical course timeline for patient’s ICU stay, illustrating major interventions, key clinical events, and selected laboratory data.

ICU Day	Major Event	Interventions	Key Labs
0	Intubation, ACLS	MV, Empiric abx, fluid, vasopressor	pH 6.81, P/F 55
3	TB PCR+, Anti-TB start	HREZ, continue MV, sedation	P/F 60, CRP 24
6	Steroid started	MPD 40 mg/d, Prone position	P/F 70
10	VAP #1, abx change	TZP+Amikacin	P/F 150, CRP 22
14	GI bleeding	EGD, transfusion, PPI	Hb 7.0
21	Tracheostomy	Decannulation protocol	P/F 180, Hb 8.8
28	VAP #2, abx change	Colistin, Tigecycline	P/F 160, CRP 10
45	Weaning, off abx/steroid	Rehab, spontaneous breathing trial	P/F 350
54	Transfer to ward	Anti-TB, rehab	Hb 11.0

## Data Availability

All relevant clinical data supporting this study are available within the article. Further data are available from the corresponding author upon request.

## References

[1] Bellani G., Laffey J.G., Pham T., Fan E., Brochard L., Esteban A., Gattinoni L., Van Haren F., Larsson A., McAuley D.F. (2016). Epidemiology, patterns of care, and mortality for patients with acute respiratory distress syndrome in intensive care units in 50 countries. JAMA.

[2] Phua J., Weng L., Ling L., Egi M., Lim C.M., Divatia J.V., Shrestha B.R., Arabi Y.M., Ng J., Gomersall C.D. (2020). Intensive care management of coronavirus disease 2019 (COVID-19): Challenges and recommendations. Lancet Respir. Med..

[3] Ranieri V.M., Rubenfeld G.D., Thompson B.T., Ferguson N.D., Caldwell E., Fan E., Camporota L., Slutsky A.S. (2012). Acute respiratory distress syndrome: The Berlin Definition. JAMA.

[4] Villar J., Blanco J., Añón J.M., Santos-Bouza A., Blanch L., Ambrós A., Gandía F., Carriedo D., Mosteiro F., Basaldúa S. (2011). The ALIEN study: Incidence and outcome of acute respiratory distress syndrome in the era of lung protective ventilation. Intensive Care Med..

[5] Laffey J.G., Bellani G., Pham T., Fan E., Madotto F., Bajwa E.K., Brochard L., Clarkson K., Esteban A., Gattinoni L. (2017). Potentially modifiable factors contributing to outcome from acute respiratory distress syndrome: The LUNG SAFE study. JAMA.

[6] Li G., Malinchoc M., Cartin-Ceba R., Venkata C.V., Kor D.J., Peters S.G., Hubmayr R.D., Gajic O. (2011). Eight-year trend of acute respiratory distress syndrome: A population-based study in Olmsted County, Minnesota. Am. J. Respir. Crit. Care Med..

[7] Chiang C.H., Lee C.H., Ho C.H., Harnod D., Liu C.K., Lin H.J., Sung F.C., Chung C.H., Kao C.H., Wu T.C. (2019). Acute respiratory distress syndrome increases short-term mortality in patients with chronic obstructive pulmonary disease: A nationwide cohort study. J. Formos. Med. Assoc..

[8] Agarwal R., Nath A., Gupta D. (2005). Noninvasive ventilation in patients with acute respiratory failure due to tuberculosis. Indian J. Med. Res..

[9] Mas A., Massip J. (2014). Noninvasive ventilation in acute respiratory failure. Int. J. Chron. Obstruct. Pulmon. Dis..

[10] Wu Y.C., Huang C.C., Wu M.S., Hsu C.W., Chen L.C., Fang C.C. (2021). Acute respiratory distress syndrome due to pulmonary tuberculosis. Tzu Chi Med. J..

[11] Ranzani O.T., Bastos L.S.L., Gelli J.G.M., Marchesi J.F., Baião F., Hamacher S., Bozza F.A. (2021). Characterisation of the first 250,000 hospital admissions for COVID-19 in Brazil: A retrospective analysis of nationwide data. Lancet Respir. Med..

[12] Theron G., Peter J., van Zyl-Smit R., Mishra H., Streicher E., Murray S., Dawson R., Whitelaw A., Hoelscher M., Sharma S. (2011). Evaluation of the Xpert MTB/RIF assay for the diagnosis of pulmonary tuberculosis in a high HIV prevalence setting. Am. J. Respir. Crit. Care Med..

[13] Lee P.L., Lee C.C., Chen W.L., Chen Y.H., Chen S.C., Lee N.Y., Ko W.C., Lee C.H., Ko P.C., Chen T.C. (2018). Tuberculous pneumonia presenting as ARDS: A case series and literature review. J. Infect. Chemother..

[14] Sharma S.K., Mohan A., Banga A., Saha P.K., Guntupalli K.K. (2006). Predictors of development and outcome in patients with acute respiratory distress syndrome due to tuberculosis. Int. J. Tuberc. Lung Dis..

[15] World Health Organization (2023). Global Tuberculosis Report. https://www.who.int/teams/global-programme-on-tuberculosis-and-lung-health/tb-reports/global-tuberculosis-report-2023.

[16] Kethireddy S., Light R.W. (2011). Acute respiratory failure and ARDS in tuberculosis. Int. J. Tuberc. Lung Dis..

[17] Brodie D., Slutsky A.S., Combes A. (2019). Extracorporeal Life Support for Adults with Respiratory Failure and Related Indications: A Review. JAMA.

[18] Cook D.J., Walter S.D., Cook R.J., Griffith L.E., Guyatt G.H., Leasa D., Jaeschke R.Z., Brun-Buisson C. (1994). Incidence of and risk factors for ventilator-associated pneumonia in critically ill patients. N. Engl. J. Med..

[19] Fan E., Brodie D., Slutsky A.S. (2018). Acute Respiratory Distress Syndrome: Advances in Diagnosis and Treatment. JAMA.

[20] Bauer T.T., Ewig S., Rodloff A.C., Müller E.E. (2006). Acute respiratory distress syndrome and pneumonia: A comprehensive review of clinical data. Clin. Infect. Dis..

[21] Kollef M.H. (1993). Ventilator-associated pneumonia: A multivariate analysis. Chest.

[22] Grudzinska F.S., Brodlie M., Scholefield B.R., Simpson A.J., Scott A., Corris P.A., Ward C., Rutherford R.M., Gould K., Lordan J. (2017). Pulmonary infections in patients with ARDS. Clin. Infect. Dis..

[23] Slutsky A.S., Ranieri V.M. (2013). Ventilator-Induced Lung Injury. N. Engl. J. Med..

[24] Phua J., Badia J.R., Adhikari N.K., Friedrich J.O., Fowler R.A., Singh J.M., Scales D.C., Stather D.R., Li A., Jones A. (2009). Has mortality from acute respiratory distress syndrome decreased over time?: A systematic review. Am. J. Respir. Crit. Care Med..

[25] Hu C.C., Hsu C.W., Kang S.P., Hsiue T.R., Yu C.J., Tsai T.H., Chen N.H., Lin M.C., Tsai C.L., Perng W.C. (2021). Factors associated with outcome of severe ARDS managed with prone positioning: Experience in a single center. J. Microbiol. Immunol. Infect..

[26] MacLaren G., Fisher D., Brodie D. (2020). Preparing for the Most Critically Ill Patients With COVID-19: The Potential Role of Extracorporeal Membrane Oxygenation. JAMA.

[27] Briel M., Meade M., Mercat A., Brower R.G., Talmor D., Walter S.D., Slutsky A.S., Pullenayegum E., Zhou Q., Cook D. (2010). Higher vs lower positive end-expiratory pressure in patients with acute lung injury and acute respiratory distress syndrome: Systematic review and meta-analysis. JAMA.

[28] Rochwerg B., Brochard L., Elliott M.W., Hess D., Hill N.S., Nava S., Navalesi P., Antonelli M., Brozek J., Conti G. (2017). Official ERS/ATS clinical practice guidelines: Noninvasive ventilation for acute respiratory failure. Eur. Respir. J..

[29] Torres A., Niederman M.S., Chastre J., Ewig S., Fernandez-Vandellos P., Hanberger H., Kollef M., Li Bassi G., Luna C.M., Martin-Loeches I. (2017). International ERS/ESICM/ESCMID/ALAT guidelines for the management of hospital-acquired pneumonia and ventilator-associated pneumonia: Guidelines for the management of hospital-acquired pneumonia (HAP)/ventilator-associated pneumonia (VAP) of the European Respiratory Society (ERS), European Society of Intensive Care Medicine (ESICM), European Society of Clinical Microbiology and Infectious Diseases (ESCMID) and Asociación Latinoamericana del Tórax (ALAT). Eur. Respir. J..

[30] Ranzani O.T., Bastos L.S.L., Gelli J.G.M., Marchesi J.F., Baião F., Hamacher S., Bozza F.A. (2021). Characterisation of the first 250,000 hospital admissions for COVID-19 in Brazil: A retrospective analysis of nationwide data. Crit. Care.

[31] Grasselli G., Zangrillo A., Zanella A., Antonelli M., Cabrini L., Castelli A., Cereda D., Coluccello A., Foti G., Fumagalli R. (2020). Baseline characteristics and outcomes of 1591 patients infected with SARS-CoV-2 admitted to ICUs of the Lombardy Region, Italy. JAMA.

[32] Needham D.M., Colantuoni E., Mendez-Tellez P.A., Dinglas V.D., Sevransky J.E., Himmelfarb C.R., Desai S.V., Shanholtz C., Brower R.G., Pronovost P.J. (2020). Lung protective mechanical ventilation and two-year survival in patients with acute lung injury: Prospective cohort study. Crit. Care Med..

[33] Gong M.N., Martin G.S. (2022). Acute respiratory distress syndrome: Clinical features, diagnosis, and management. Crit. Care Clin..

[34] Krag M., Perner A., Wetterslev J., Wise M.P., Borthwick M., Bendel S., McArthur C., Cook D., Nielsen N., Pelosi P. (2015). Stress ulcer prophylaxis in the intensive care unit: An international survey of 97 units in 11 countries. Acta Anaesthesiol. Scand..

[35] Lim J., Lee S., Kim Y.J., Lee Y.J., Lee J., Lee J.H., Jung H.B., Kim M.S., Lim S.Y., Park S. (2021). Outcomes of severe ARDS patients managed with high-frequency oscillatory ventilation. Crit. Care.

[36] Mokhlesi B., Tulaimat A., Evans A.T., Wang Y. (2002). Association between IL-6 levels and outcome in ARDS. Chest.

[37] Tobin M.J. (2020). Basing Respiratory Management of COVID-19 on Physiological Principles. Am. J. Respir. Crit. Care Med..

[38] Kuperminc E., Heming N., Carlos M., Annane D. (2023). Corticosteroids in ARDS. J. Clin. Med..

[39] National TB Program Taiwan (2023). Annual Report. https://www.cdc.gov.tw/En/File/Get/vM5svuwyGvbljFbKDPQlTw.

